# Is the blood pressure response to exercise associated with the motor unit firing patterns of the exercised muscle?

**DOI:** 10.1113/EP092462

**Published:** 2025-01-03

**Authors:** Shigehiko Ogoh

**Affiliations:** ^1^ Department of Biomedical Engineering Toyo University Asaka Saitama Japan

**Keywords:** blood pressure, exercise, muscle

1

Arterial blood pressure (ABP) increases during exercise to support the elevated muscle metabolism required for physical activity. This response is particularly pronounced in elderly individuals, those with cardiovascular diseases such as hypertension, and even some healthy individuals (Kunimatsu et al., 2024). Notably, such an exaggerated ABP response during exercise is associated with an increased risk of damage to cerebral and cardiac vasculature, especially in these populations. Despite these concerns, the mechanisms underlying individual differences in ABP responses to exercise remain poorly understood. ABP during exercise is influenced by the force generated during physical activity, which is regulated by the neuromuscular system. This regulation involves factors such as motor unit firing rates, motor unit recruitment thresholds and the interplay between these elements. Additionally, these factors play a crucial role in determining the efficiency of microvascular unit perfusion during exercise. These findings suggest that the motor unit firing pattern may be a key determinant of ABP response to exercise. Given this background, in this issue of *Experimental Physiology*, Takeda et al. (2025) report differences in the motor unit firing patterns during exercise between normotensive, treated hypertensive and untreated hypertensive individuals. They also examined the effects of these differences in motor unit firing patterns on post‐exercise ABP responses.

The authors found that in treated hypertensive and normotensive individuals, firing rates of larger motor units were lower than those of smaller motor units, while in untreated hypertensive individuals, firing rates of larger motor units were similar to those of smaller ones. This suggests that motor unit firing patterns differ across cardiovascular conditions. Additionally, they reported that the firing rates and slope of larger motor units were positively correlated with changes in systolic blood pressure (SBP) from rest to post‐exercise in hypertensive individuals. This implies a link between exercise‐induced motor unit firing patterns and cardiovascular response. However, post‐exercise ABP responses did not directly reflect the ABP response during exercise, making it challenging to determine the effect of motor unit firing patterns on ABP in this study. Also, the negative change in SBP in hypertensive individuals from baseline to post‐exercise may involve several physiological factors and should not be directly tied to exercise‐induced ABP changes. Nevertheless, it is essential to confirm whether their hypothesis holds true within the context of this study.

Fortunately, previous studies (Currie et al., 2025; Lacy et al., 2015) have reported ABP responses to exercise in hypertensive individuals, allowing speculation on how motor unit firing patterns may influence ABP responses. It is well‐established that hypertensive patients have a larger ABP response to exercise compared to normotensive individuals (Currie et al., 2025). In this context, the result of this study, showing that the firing rates of large motor units—which increase intramuscular pressure and peripheral vascular resistance (Osada et al., 2015)—were higher in untreated hypertensive individuals, provides a possible physiological mechanism of the exaggerated ABP response during exercise in hypertensive individuals. In other words, the hypertensive condition may modify the motor units firing pattern, which consequently leads to a higher ABP response to exercise. Interestingly, treated hypertensive individuals did not show an increase in the firing rates of large motor units. It has been reported that treated hypertensive individuals do not exhibit an exaggerated ABP response during exercise with angiotensin receptor blockade (Lacy et al., 2015), suggesting that the normalization of the firing rates of large motor units in treated hypertensive individuals may be linked to the disappearance of the exaggerated ABP response during exercise. Therefore, even in hypertensive individuals, the ABP response to exercise may be modulated by changes in the motor unit firing pattern.

These findings, regarding the parallel change in both the motor unit firing pattern and the ABP response to exercise observed in the three groups of this study, suggest that motor unit firing patterns determine the ABP response to exercise, and vice versa. However, the previous findings that activating larger motor units increases intramuscular pressure and peripheral vascular resistance (Osada et al., 2015) support the assumption that motor unit firing patterns determine the ABP response to exercise. However, this estimation is based on the previous findings, and a new study is necessary to identify the effect of motor unit firing patterns on the ABP response to exercise by directly measuring motor unit firing patterns alongside ABP during exercise. Moreover, questions remain about why hypertension and angiotensin receptor blockade medications modify motor unit firing patterns. The mechanisms underlying this phenomenon are not fully understood, but one possible explanation is oxidative stress, which has been shown to cause motor neuron injury. Another potential factor is muscle metabolism. Both oxidative stress and muscle metabolism, which are influenced by hypertensive conditions, may contribute to alterations in motor unit firing patterns. Further research is needed to confirm these physiological mechanisms, as they remain speculative at this stage. Such investigations could provide valuable insights for developing effective exercise therapies for hypertensive patients.

## AUTHOR CONTRIBUTIONS

Sole author.

## CONFLICT OF INTEREST

The author declares no conflicts of interest.

## FUNDING INFORMATION

No funding was received for this work.
